# Identification of candidate genes for key fibre‐related QTLs and derivation of favourable alleles in *Gossypium hirsutum* recombinant inbred lines with *G. barbadense* introgressions

**DOI:** 10.1111/pbi.13237

**Published:** 2019-09-20

**Authors:** Furong Wang, Jingxia Zhang, Yu Chen, Chuanyun Zhang, Juwu Gong, Zhangqiang Song, Juan Zhou, Jingjing Wang, Chengjie Zhao, Mengjia Jiao, Aiying Liu, Zhaohai Du, Youlu Yuan, Shoujin Fan, Jun Zhang

**Affiliations:** ^1^ Key Laboratory of Cotton Breeding and Cultivation in Huang‐Huai‐Hai Plain Ministry of Agriculture Cotton Research Center of Shandong Academy of Agricultural Sciences Jinan China; ^2^ College of Life Sciences Shandong Normal University Jinan China; ^3^ State Key Laboratory of Cotton Biology Key Laboratory of Biological and Genetic Breeding of Cotton Ministry of Agriculture Institute of Cotton Research Chinese Academy of Agricultural Sciences Anyang China

**Keywords:** *Gossypium hirsutum* L., fibre quality and yield, stable QTLs, candidate gene, introgression, *G. barbadense *L.

## Abstract

Fine mapping QTLs and identifying candidate genes for cotton fibre‐quality and yield traits would be beneficial to cotton breeding. Here, we constructed a high‐density genetic map by specific‐locus amplified fragment sequencing (SLAF‐seq) to identify QTLs associated with fibre‐quality and yield traits using 239 recombinant inbred lines (RILs), which was developed from LMY22 (a high‐yield *Gossypium hirsutum*L. cultivar) × LY343 (a superior fibre‐quality germplasm with *G*. *barbadense*L. introgressions). The genetic map spanned 3426.57 cM, including 3556 SLAF‐based SNPs and 199 SSR marker loci. A total of 104 QTLs, including 67 QTLs for fibre quality and 37 QTLs for yield traits, were identified with phenotypic data collected from 7 environments. Among these, 66 QTLs were co‐located in 19 QTL clusters on 12 chromosomes, and 24 QTLs were detected in three or more environments and determined to be stable. We also investigated the genomic components of LY343 and their contributions to fibre‐related traits by deep sequencing the whole genome of LY343, and we found that genomic components from *G. hirsutum* races (which entered LY343 via its *G*. *barbadense* parent) contributed more favourable alleles than those from *G. barbadense*. We further identified six putative candidate genes for stable QTLs, including *Gh_A03G1147* (*GhPEL6*), *Gh_D07G1598* (*GhCSLC6*) and *Gh_D13G1921* (*GhTBL5*) for fibre‐length QTLs and *Gh_D03G0919* (*GhCOBL4*), *Gh_D09G1659* (*GhMYB4*) and *Gh_D09G1690* (*GhMYB85*) for lint‐percentage QTLs. Our results provide comprehensive insight into the genetic basis of the formation of fibre‐related traits and would be helpful for cloning fibre‐development‐related genes as well as for marker‐assisted genetic improvement in cotton.

## Introduction

Cotton (*Gossypium* spp.) is the leading natural fibre crop in the world. Among the domesticated *Gossypium* species, *G. hirsutum* is the most widely cultivated around the world and dominates modern worldwide cotton production due to its high lint yield and broad adaptability (Rahman *et al*., 2012; Wang *et al*., 2017b; Zhang *et al*., 2015b). Because of the limited genetic diversity within cultivated upland cotton (May *et al*., 1995; Tyagi *et al*., 2014), simultaneous improvement of fibre quality and yield via conventional breeding methods has been difficult. However, long‐term interspecific hybridization has developed a large amount of germplasm with obviously improved traits, such as fibre quality and/or biotic or abiotic tolerances, that contains introgressed components from other *Gossypium* species (Abdelraheem *et al*., 2018; Bell *et al*., 2014, 2015; Iqbal *et al*., 2015; Keerio *et al*., 2018; Mammadov *et al*., 2018; Wang *et al*., 2017a; Xu *et al*., 2012; Zhang *et al*., 2011; Zhou *et al*., 2003), especially from cultivated *G. barbadense* varieties (Chen *et al*., 2018; Wang *et al*., 2011a, 2013, 2019). Unfortunately, a major problem in using these germplasm resources is the negative correlation between fibre quality and lint yield (Scholl and Miller, 1976; Zhou *et al*., 2003). Breeding practice has shown that this correlation is difficult to overcome depending on only conventional breeding methods.

In addition to endowing genetically modified organisms (GMOs) with the desired new traits, plant biotechnology provides a new approach for the precise identification and effective utilization of the beneficial genes or genomic components contained in various genetic resources for crop genetic improvement (Grandillo, 2014; Zamir, 2001, 2008). Simultaneous enhancement of fibre quality and lint yield by identification and utilization of introgressed genomic components, especially *G. barbadense* introgressions, was recently reported (Chen *et al*., 2018; Fang *et al*., 2017a; Guo *et al*., 2018; Li *et al*., 2019; Wang *et al*., 2019; Wen *et al*., 2018). QTL mapping is one of the approaches used to find tightly linked markers for marker‐assisted selection (MAS). Many studies have shown that the number and precision of QTLs detected could increase with increasing marker density in genetic maps (Jamshed *et al*., 2016; Negro *et al*., 2019). With the development of high‐throughput sequencing technology, SNP markers have been widely applied to construct high‐density genetic maps and to map QTLs, due to their extensive and intensive distribution throughout genomes. Specific‐locus amplified fragment sequencing (SLAF‐seq) has been widely used to develop SNP markers for constructing high‐density genetic maps and mapping QTLs in many crops, such as soybean (*Glycine max*) (Li *et al*., 2014, 2017), peanut (*Arachis hypogaea*) (Hu *et al*., 2018), cucumber (*Cucumis sativus*) (Wei *et al*., 2014; Xu *et al*., 2014), sesame (*Sesamum indicum*) (Mei *et al*., 2017; Zhang *et al*., 2013) and other crops (Tao *et al*., 2017; Wei *et al*., 2017; Zhang *et al*., 2017a). SLAF‐based SNP markers have also been used in cotton to construct high‐density genetic maps and identify QTLs for *Verticillium* wilt resistance (Shen *et al*., 2017; Li *et al*., 2017), boll weight (Zhang *et al*., 2016) and fibre‐quality‐related traits (Jia *et al*., 2018).

In our previous study, we identified *G. barbadense* introgressions in the superior fibre‐quality germplasm LY343 and found that most of favourable alleles for fibre quality were related to its introgressed genetic components (Chen *et al*., 2018; Wang *et al*., 2011a, 2013, 2016). However, similar to most genetic maps of *Gossypium* species (Guo *et al*., 2007; Yu *et al*., 2011), all our mapping work was based on SSR markers. The lower density of SSR markers severely limited mapping efficiency and precision.

In this study, we integrated SLAF‐based SNP and SSR markers to construct a high‐density genetic map using 239 RILs, and we subsequently identified stable QTLs, epistatic QTLs and QTL clusters associated with fibre‐quality and yield traits across 7 environments. We also investigated the genetic constitution of LY343 and analysed the contributions of introgressions to fibre‐related traits by resequencing the whole genome of LY343. Furthermore, we combined DNA resequencing, RNA‐seq and RT‐qPCR to identify candidate genes for stable QTLs. Our results will be beneficial for understanding the genetic basis of positive and/or negative correlations between fibre quality and lint yield for simultaneously improving the yield and quality of upland cotton via MAS and/or genetic manipulation.

## Results

### SLAF sequencing and SLAF markers

*Hae*III and *Ssp*I were chosen to construct SLAF libraries after the pilot experiment, and 623.61 M paired‐end 80‐bp reads were finally generated (Table S1). The high‐quality bases with Q30 (base calling error at the 0.001 level) were 80.40% of the total, and the average GC (guanine and cytosine) content was 38.12%. A total of 432 870 SLAFs were developed after read clustering, 222 931 SLAFs in the A sub‐genome and 115 411 SLAFs in the D sub‐genome while the remaining SLAFs located in scaffolds. The average sequencing depths of these SLAFs were 37.4‐fold for the parents and 6.08‐fold for each line of the RIL population. Among the developed SLAFs, 25 280 were polymorphic, yielding a polymorphism rate of 5.84% (Figure S1, Table S2); the polymorphism rate of Chr. D07 was the highest, followed by Chr. A02. All the polymorphic SLAFs were used to genotype the mapping parents, and 15 546 were found to be homozygous in each parent and retained for further analysis. Then, the SLAFs with average sequencing depths < 10‐fold in the parents and with significant segregation distortion (*P* < 0.001) were filtered out. Finally, 3749 SLAFs were defined as high‐quality markers and used to construct the final genetic map.

### Construction and quality assessment of the genetic map

A total of 3749 high‐quality SLAF markers together with 226 polymorphic SSR markers identified in our previous study (Wang *et al*., 2016) were integrated to construct a high‐density genetic linkage map (LG). The pairwise modified logarithm of odds (MLOD) scores of the markers were calculated and used to construct the linkage map. A total of 3755 markers, including 3556 SLAFs and 199 SSR markers, were finally mapped to the LGs, spanning a total distance of 3426.57 cM with an average marker interval of 1.5 cM (Table S3, Table S4). The longest LG was Chr. D07, which spanned 194.67 cM and contained 490 markers with an average 0.4‐cM marker interval. The shortest LG was Chr. A06, which was 40.62 cM in length with 24 markers and a 1.69‐cM marker interval. Chr. A02 harboured the greatest marker number of 788, followed by Chr. D07. Among the mapped markers, more than half (1951) showed significant segregation distortion, and 73.8% of them were biased towards the female parent, LMY22. Chr. A02, on which all the markers except one were biased, had the largest number of segregation distortion markers (SDMs), followed by Chr. D07, on which 393 out of 490 markers were biased. Notably, SDRs (segregation distortion regions) are usually located in introgressed chromosomal regions.

Collinearity analysis of each LG with the cotton reference genome was conducted to evaluate the quality of the genetic map (Figure S1, Table S5). The average Spearman coefficient was 0.628, suggesting that the genetic map had relatively high collinearity with the cotton reference genome. Chr. A09, D01, D05 and D12 showed higher collinearity (Spearman coefficient > 0.9), but Chr. D02 had lower consistency, with a Spearman coefficient of only 0.0135.

### Phenotypic assessment and correlation analysis

The two parents, LMY22 and LY343, showed significant (*P *<* *0.05) or highly significant (*P *<* *0.001) differences for all the examined traits except for fibre elongation (FE) and boll number (BN) (Table S6). The *G*. *hirsutum* parent, LMY22, had higher values for yield‐related traits, such as boll weight (BW) and lint percentage (LP), while the introgression line (IL) parent, LY343, had higher values for favourable fibre‐quality traits, such as fibre length (FL), fibre length uniformity (FU), fibre strength (FS) and fibre micronaire (FM). Among the RIL population, FL, FS, FM, LP and BW showed relatively high heritability and were normally distributed in most environments. However, FU, FE and BN, which had low heritability, were normally distributed in only a few environments.

A correlation analysis of all examined traits was conducted and is presented in Table S7. Highly significant correlations were observed among fibre‐quality traits and yield traits. A strong positive correlation occurred among FL, FU and FS, but a strong negative correlation existed between FL and FM as well as FS and FM. Among yield traits, BW was significantly negatively correlated with BN, with no significant correlations between BW, BN and LP. Regarding yield and fibre‐quality traits, LP were significantly negatively correlated with FL and FS but positively correlated with FM, FU and FE.

### QTL mapping

**Figure 1:**
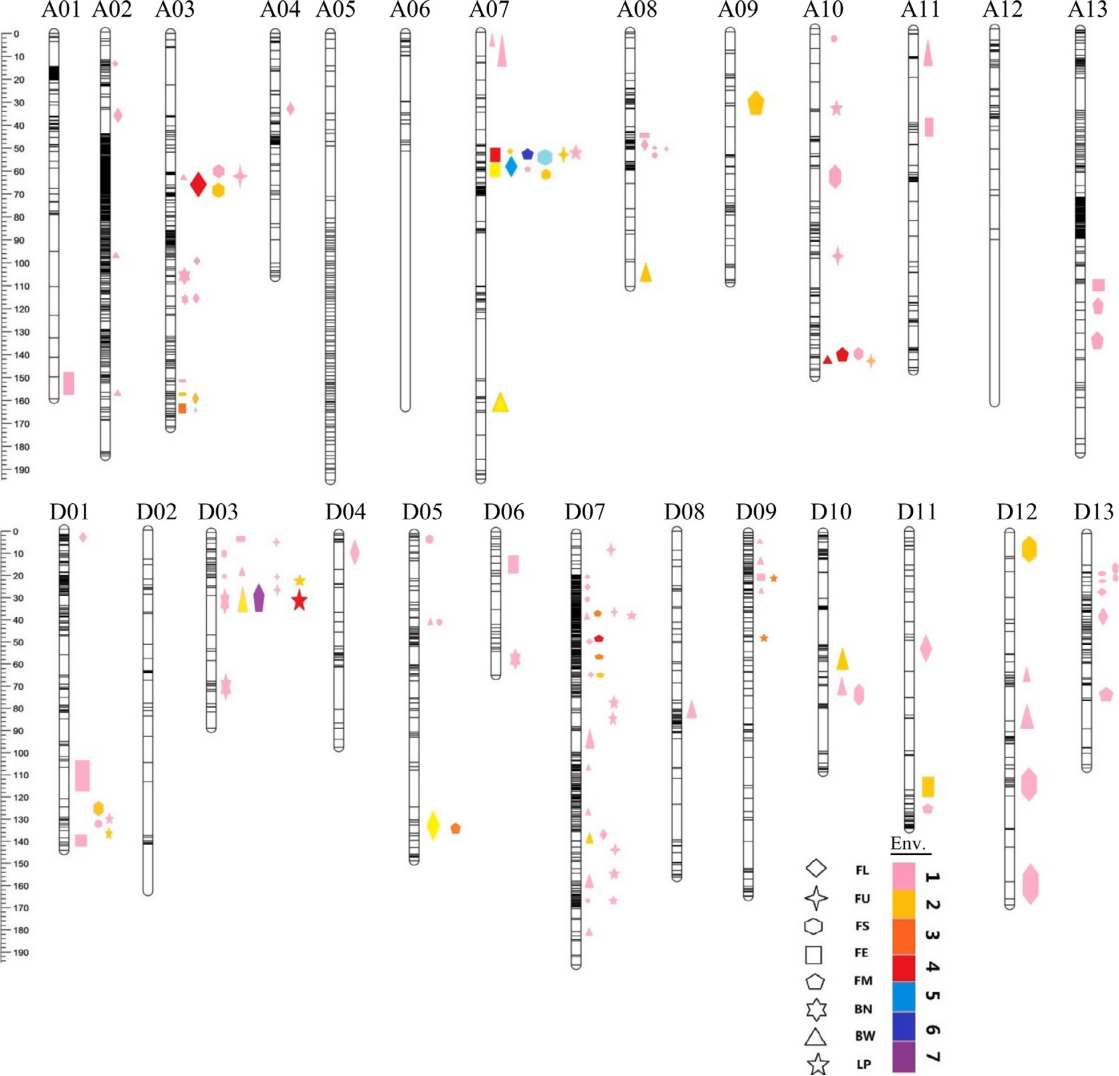
QTLs and QTL clusters identified for fibre‐quality and yield traits. The ruler on the left of chromosomes indicates the genetic distance (cM). The patterns of different shapes represent different phenotypes: FL, fibre length; FU, fibre‐length uniformity; FS, fibre strength; FE, fibre elongation; FM, fibre micronaire; BN, boll number; BW, boll weight; and LP, lint percentage. The different colours of patterns represent 1–7 environments which QTL can be detected in just as the corresponding number on the right of the coloured bar in the legend.

A total of 104 QTLs, including 67 for fibre quality and 37 for yield, were identified, explaining 3.40%–37.66% of the observed phenotypic variation (Figure 1, Table S8). The high‐quality parent, LY343, contributed the majority (44/67, 65.7%) of favourable alleles for fibre‐quality QTLs, while the high‐yield parent, LMY22, contributed the majority (27/37, 73.0%) of favourable alleles for yield QTLs. The number of fibre‐quality QTLs distributed in the A sub‐genome was similar to that in the D sub‐genome (31/36). However, for yield‐related QTLs, the number distributed in the D sub‐genome was two times as much as that in the A sub‐genome (25/12).

Twenty‐four of the QTLs were detected in three or more environments and determined tobe stable (Table S9). Six of the stable QTLs explaining more than 10% of the observed phenotypic variation in some of the environments were identified as major QTLs, that is *qFS‐A07‐1*,* qFE‐A03‐1*,* qFM‐A07‐1, qFM‐D07‐2, qBW‐A10‐1* and *qLP‐D03‐1*. For fibre‐quality traits, 15 stable QTLs were identified, including 4 for FL, 1 for FU, 3 for FS, 2 for FE and 5 for FM, and for yield traits, 9 stable QTLs were identified, including 4 each for LP and BW and 1 for BN. Chromosome A07 contained the largest number (4) of stable QTLs for fibre‐quality traits, while D03 contained the largest number (3) of stable QTLs for yield traits; these results suggested that important loci associated with fibre quality and yield were located on these two chromosomes.

### Epistatic interactions of QTLs

Interactions among loci have a substantial contribution to the phenotypic variation of quantitative traits (Carlborg and Haley, 2004). Therefore, we further identified epistatic interactions focusing on stable QTLs. A total of 44 digenic epistatic interactions with additive × additive (AA) effects were detected, and a majority of these interactions occurred between stable QTLs and genetic background loci (Table S10). Only three pairs of epistatic interactions were detected between stable QTLs and significant QTLs, that is *qFS‐A03‐1* and *qFS‐D12‐1*,* qFS‐A07‐1* and *qFS‐D01‐1*,* qLP‐D01‐1* and *qLP‐D07‐2*, among which *qFS‐A07‐1* and *qFS‐D01‐1* are a pair of stable QTLs. Many stable QTLs, such as *qFS‐A03‐1*,* qFS‐A07‐1* and *qLP‐D01‐1*, interacted with more than three loci on different chromosomes, and the reverse was also detected, as *qFU‐A10‐3* was an interaction target of three loci on different chromosomes.

### QTL clusters associated with fibre‐quality and yield traits

Two or more QTLs for various traits co‐located within an approximately 10 cM region were defined as a QTL cluster. A total of 19 QTL clusters were identified; these clusters comprised 65 QTLs and were distributed on 12 chromosomes (Figure 1, Table S11). Chr. D07 had the greatest number of QTL clusters (5 clusters). Eleven of the QTL clusters contained at least one stable QTL, suggesting that these regions include key genes associated with fibre‐quality or yield traits. Twelve QTL clusters were associated with not only fibre quality but also yield components, and five QTL clusters (Cluster‐A08‐1, Cluster‐D05‐1, Cluster‐D07‐2, Cluster‐D11‐1 and Cluster‐D13‐1) were associated with only fibre‐quality traits, while two QTL clusters (Cluster‐D07‐3 and Cluster‐D07‐5) were associated with only yield traits. Generally, within the same QTL cluster, fibre‐quality traits had the same genetic effect, but fibre‐quality and yield traits had opposite genetic effects. However, QTLs for BW and LP, the two main components of yield traits, had the same direction of effect within two QTL clusters (Cluster‐D07‐1 and Cluster‐D09‐1), while opposite genetic effects existed in three QTL clusters (Cluster‐D03‐1, Cluster‐D07‐3 and Cluster‐D07‐5).

### Genomic constitution of LY343 and contribution of introgressions to fibre quality

**Figure 2:**
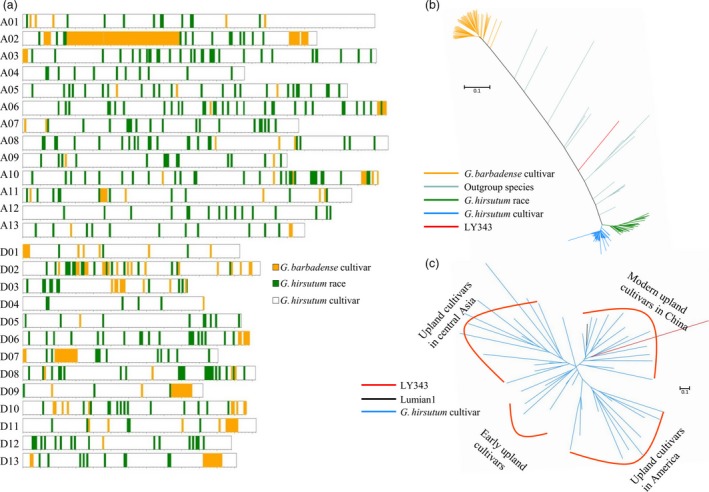
Genetic constitution of LY343. (a) Introgression chromosomal segments in LY343. (b) A phylogenetic tree of chromosome A02 which has the most *G. barbadense* introgressions. (c) Phylogenetic relationship between LY343 and *G. hirsutum* cultivars. LY343 (red) was classed into the same subgroup as modern upland cultivars in China including Lumian 1 (black).

LY343 is a high‐fibre‐quality germplasm, which originated from a natural hybridization of *G. hirsutum* and *G. barbadense* followed by artificial selection for several generations. The main phenotypic characteristics of LY343 were recovered to those of *G. hirsutum*. However, the pedigree of LY343 remains unclear. To analyse the contribution of the exotic introgressed segments to fibre‐related traits, we deep‐sequenced the whole genome of LY343. By combining this sequence with the genomic data of 147 cotton accessions (Fang *et al*., 2017a), neighbour‐joining (NJ) trees of every 500‐Kb genomic sequence on the 26 cotton chromosomes were built to detect the introgressed genetic components in LY343. The results showed that 94.70% of the genomic sequence of LY343 was from *G. hirsutum* cultivars, 3.88% from *G. hirsutum* races and 1.42% from *G. barbadense* (Table S12, Figure 2a and Figure S2), consistent with the phenotypic characteristics of LY343. Chromosome A02 has the most introgression components from *G. barbadense* (46.18%), followed by D10 (13.51%) and D04 (11.73%) (Table S12, S13); chromosome D01 has the most introgressions from *G. hirsutum* races (20.66%), followed by D07 (8.91%) and A10 (8.70%) (Table S12, S14). The introgressed *G. hirsutum* race segments in LY343 may be due to the early introgression from *G. hirsutum* races into *G. barbadense* (Fang *et al*., 2017a). We further analysed the genomic components from *G. hirsutum* cultivars in LY343 together with 52 upland cultivars by constructing a phylogenetic tree and found that LY343 was clustered into the same subgroup as modern upland cultivars in China (Figure 2c).

Among 67 fibre‐quality QTLs, the favourable alleles of 26 QTLs were derived from the introgression segments, and 11 (73%) of the 15 stable QTLs for fibre quality were located in the introgressed genomic region (Table S15). For two important fibre‐quality parameters with high heritability, FL and FS, 3 (*qFL‐A03‐1*,* qFL‐A07‐1* and *qFL‐D13‐1*) of the 4 stable fibre‐length QTLs and 2 (*qFS‐A03‐1* and *qFS‐A07‐1*) of the 3 stable fibre‐strength QTLs were located in the introgressed chromosomal segments. Among the 17 QTL clusters associated with fibre quality, 10 clusters were located in or around the introgressed regions (Table S16). Surprisingly, among 26 QTLs contributing to fibre quality, the majority (20/26, 76.92%) of QTLs were located in the genomic regions introgressed from *G. hirsutum* races instead of *G. barbadense*, and important QTL clusters associated with fibre quality, such as Cluster‐A03‐1, containing two stable QTLs (*qFL‐A03‐1* and *qFS‐A03‐1*), and Cluster‐A07‐1, containing four stable QTLs (*qFL‐A07‐1*,* qFS‐A07‐1*,* qFM‐A07‐1* and *qFE‐A07‐1*), explaining 6.4%–37.66% of the phenotypic variation in fibre quality, overlapped with the introgressed segments from *G. hirsutum* races.

### Functional annotation of candidate genes in QTL clusters

We examined the genes in the confidence intervals of 17 QTL clusters associated with fibre‐related traits and identified 2441 genes in these QTL intervals (Table S17). Among these genes, 1841 were expressed in the developing fibres of the mapping parents. Gene Ontology (GO) analysis based on an *Arabidopsis thaliana* database showed that 951 genes were annotated in 164 terms (Table S18), of which 36 genes were annotated in cell wall organization or biogenesis, 36 genes in macromolecule localization and 11 in microtubule‐based process (Figure S3).

**Figure 3:**
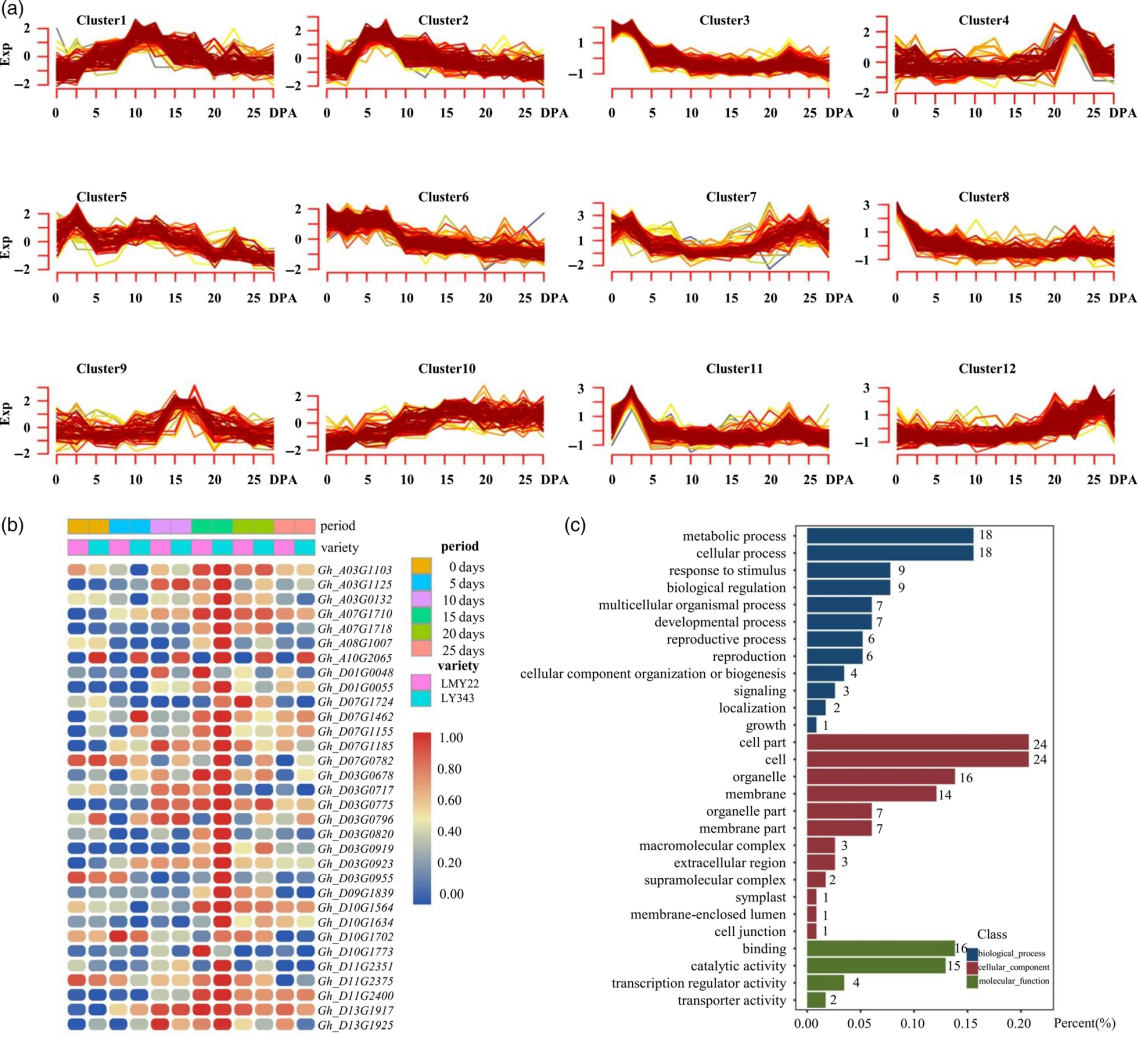
Expression patterns of candidate genes in 17 QTL clusters in LY343. (a) Expression profile of candidate genes. (b) Heat map of genes in expression profile of Cluster 9 which highly expressed in fibre at 15 DPA. (c) Go annotation of genes in Cluster 9.

Kyoto Encyclopedia of Genes and Genomes (KEGG) analysis showed that 343 genes were enriched in 128 pathways (Table S19, Figure S3), including fibre‐development‐related pathways, such as plant hormone signal transduction (18 genes), amino sugar and nucleotide sugar metabolism (13 genes), starch and sucrose metabolism (13 genes) and fatty acid metabolism (14 genes). The expression profiles of all 1841 genes at different fibre development stages were further analysed and divided into 12 clusters (Table S20, Figure 3a). Genes in Cluster 3 (Figure S4), Cluster 2 and Cluster 6 (Figure S5 and S6), which were highly expressed at 0 days post‐anthesis (DPA), 5 DPA and both stages, may be associated with fibre initiation and elongation. Genes in Cluster 1 (Figure S7) and Cluster 9 (Figure 3b and c), which were highly expressed at 10 DPA and 15 DPA, respectively, may be related to fibre cell elongation and cell wall thickening. Genes in Cluster 10, which are highly expressed in fibres from 10 DPA to 25 DPA, may play important roles in the later stages of fibre development, including fibre cell elongation, cell wall thickening and even fibre maturation processes (Figure S8).

### Identification of candidate genes associated with stable QTLs

**Figure 4:**
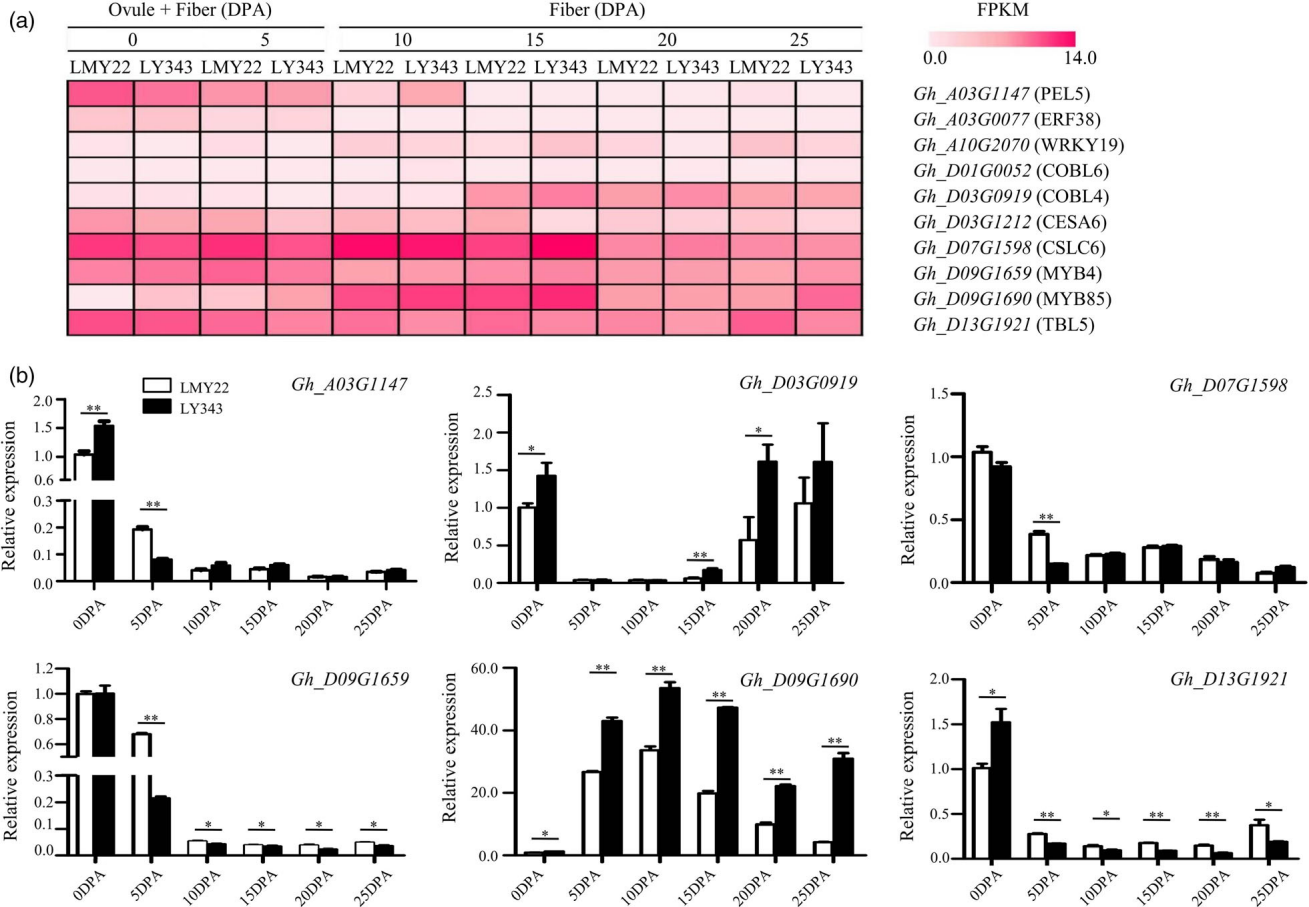
Expression patterns of fibre‐development‐related genes in different development stages. 0 and 5 DPA: mixture of ovule and fibre; 10, 15 and 25 DPA: fibre. (a) Heat map for expression patterns of candidate genes for stable QTLs at different fibre development stages. The gradation of colours represents different FPKM values. (b) Expression analysis of six candidate genes associated with fibre length and lint percentage by RT‐qPCR. * and ** indicate significantly differential expression at 0.05 and 0.01 level.

We examined genes located in the 99% confidence intervals of the stable QTLs. When two or more stable QTLs for various traits overlapped in the same QTL cluster, we focused on the QTL with higher trait heritability, such as those for FL, BW and LP. Because of the relatively high mapping resolution, 8 stable QTLs with relatively small genomic intervals were left for identifying candidate genes (Table S21). We further examined candidate gene expression at various fibre developmental stages in LMY22 and LY343 using RNA‐seq data (Figure 4a), verified the results by RT‐qPCR (Figure 4b), and finally identified 6 candidate genes associated with 5 stable QTLs.

*qFL‐A03‐1*, detected in four environments, overlapping with *qFS‐A03‐1*,* qFU‐A03‐1* and *qBW‐A03‐1* in Cluster‐A03‐1, was positioned at 75.3–84.5 Mb on A03, where a *pectate lyase* gene (*PEL6*,* Gh_A03G1147*), responsible for degrading pectin (Sun *et al*., 2018), was located. The RNA‐seq data and RT‐qPCR results indicated that *Gh_A03G1147* was relatively highly expressed during the earlier stages (0 and 5 DPA) of fibre development and had significantly higher expression in LMY22 than in LY343. However, the resequencing data revealed no SNP or indel variation in the coding or promoter region of this gene.

*qFL‐D07‐1*, detected in four environments, overlapping with *qFM‐D07‐1*,* qFM‐D07‐2*,* qBW‐D07‐1* and *qLP‐D07‐1* in Cluster‐D07‐1, was mapped to a 0.99‐Mb interval on D07, where *CSLC6* (*Gh_D07G1598*), a member of the cellulose‐synthase‐like C subfamily responsible for synthesizing the xyloglucan backbone chain (Liepman and Cavalier, 2012), was located. *Gh_D07G1598* (*GhCSLC6*) had significantly higher expression at the early stage of fibre development (5 DPA) in LMY22 than in LY343.

*qFL‐D13‐1*, detected in three environments and overlapping with *qFS‐D13‐1*, and *qFM‐D13‐1* in Cluster‐D13‐1, was mapped to a 1.36‐Mb interval on D13, where *TBL5* (*TRICHOME BIREFRINGENCE‐LIKE 5*,* Gh_D13G1921*), a member of the *TBL* gene family, was located. The resequencing data revealed one nonsynonymous SNP mutation (G + 985 → A + 985) in exon 5 of *TBL5* (*Gh_D13G1921*: exon 5: c.G985A: p.V329I) and one indel variation (‐450AGGG AGGG‐458) in the promoter region of *TBL5* (Table S22). RNA‐seq data and RT‐qPCR indicated that *Gh_D13G1921* was significantly more highly expressed in developing fibres from 0 to 25 DPA in LY343 than in LMY22.

*qLP‐D03‐1* was detected in all seven environments and explained 10.44%–33.31% of the phenotypic variation, overlapping with *qFM‐D3‐1*,* qFU‐D03‐1*,* qBN‐D03‐2* and *qBW‐D03‐1* in Cluster‐D03‐2, suggesting that key genes controlling fibre development reside in this QTL region. Therefore, we also examined the candidate genes for *qLP‐D03‐1,* as it was mapped to a relatively large interval of 19.42 Mb on D03. *CESA6* (*cellulose synthase 6*,* Gh_D03G1212*), which is involved in primary cell wall synthesis (Liepman and Cavalier, 2012), and *COBL4* (*COBRA‐like 4*,* Gh_D03G0919*), which is involved in secondary cell wall synthesis (Liu *et al*., 2013), were located in this QTL region. The resequencing data revealed a 249‐bp insertion in intron 6 of *Gh_D03G0919* (D03: 31267970–31267987) and a 1200‐bp duplication in exon 1 of *Gh_D03G1212* (D03: 39195801–39197000). The RNA‐seq data and RT‐qPCR results showed that *Gh_D03G0919* had higher expression in thickening fibres from 15 to 25 DPA and was differentially expressed in LY343 and LMY22. However, *Gh_D03G1212* showed no obvious expression differences at any stage of fibre development or between LMY22 and LY343.

*qLP‐D09‐1*, detected in three environments, overlapping with *qBW‐D09‐1* and *qFE‐D09‐1* in Cluster‐D09‐1, was mapped to a 0.85‐Mb interval on D09, where *MYB 4* (*Gh_D09G1659*), which negatively regulates lignin synthesis (Jin *et al*., 2000), and *MYB 85* (*Gh_D09G1690*), which positively regulates lignin synthesis (Wang *et al*., 2011b; Zhong *et al*., 2008), were located. The resequencing data revealed one nonsynonymous SNP mutation (C + 4 → G + 4) in exon 1 of *MYB 4* (*Gh_D09G1659*: exon 1: c.C4G: p.Q2E) and one indel variation (‐248AATATATATATA‐259) in the promoter region of *MYB4*. One indel variation (‐68CAAAAAAAAAA‐58) was also detected in the promoter region of *MYB85*. The RNA‐seq data showed that *Gh_D09G1659* was highly expressed during all stages of fibre development, while *Gh_D09G1690* was expressed at lower levels in 0‐ and 5‐DPA fibres and increased dramatically at 10 and 15 DPA, followed by an abrupt decrease at 20 DPA. RT‐qPCR indicated that *Gh_D09G1659* had significantly higher expression during the whole process of fibre development in LMY22, except at 0 and 15 DPA, whereas *Gh_D09G1690* had significantly higher expression in LY343 fibres from 5 to 25 DPA.

*MYB4* and *MYB85*, the homologous genes of *Gh_D09G1659* and *Gh_D09G1690* in *Arabidopsis*, act as an inhibitor and an activator, respectively, to regulate the expression of genes related to lignin synthesis (Jin *et al*., 2000; Zhong *et al*., 2008; Zhong and Ye, 2014). We examined the expression patterns of such genes downstream of *Gh_D09G1659* and *Gh_D09G1690* by RT‐qPCR (Figure S9). As opposed to *Gh_A09G1659* (*MYB4*), the expression of *GhCHSY* (*Gh_D09G0001*) was up‐regulated during the elongation and secondary wall thickening period and was higher in LMY22 than in LY343, indicating potential negative regulation. However, the expression patterns of *GhCYP73* (*Gh_A13G2057*) and *GhCHS1* (*Gh_D10G1429*) were similar to *GhMYB4* (*Gh_A09G1659*). Additionally, the expression levels of *Gh4CL2* (*Gh_A10G0456*), *GhC4H* (*Gh_A10G1590*) and *GhCCoAOMT* (*Gh_A04G1207*) in LY343 were higher than those in LMY22. In particular, the expression of *GhCCoAOMT* was significantly increased at the later stage of fibre development.

**Figure 5:**
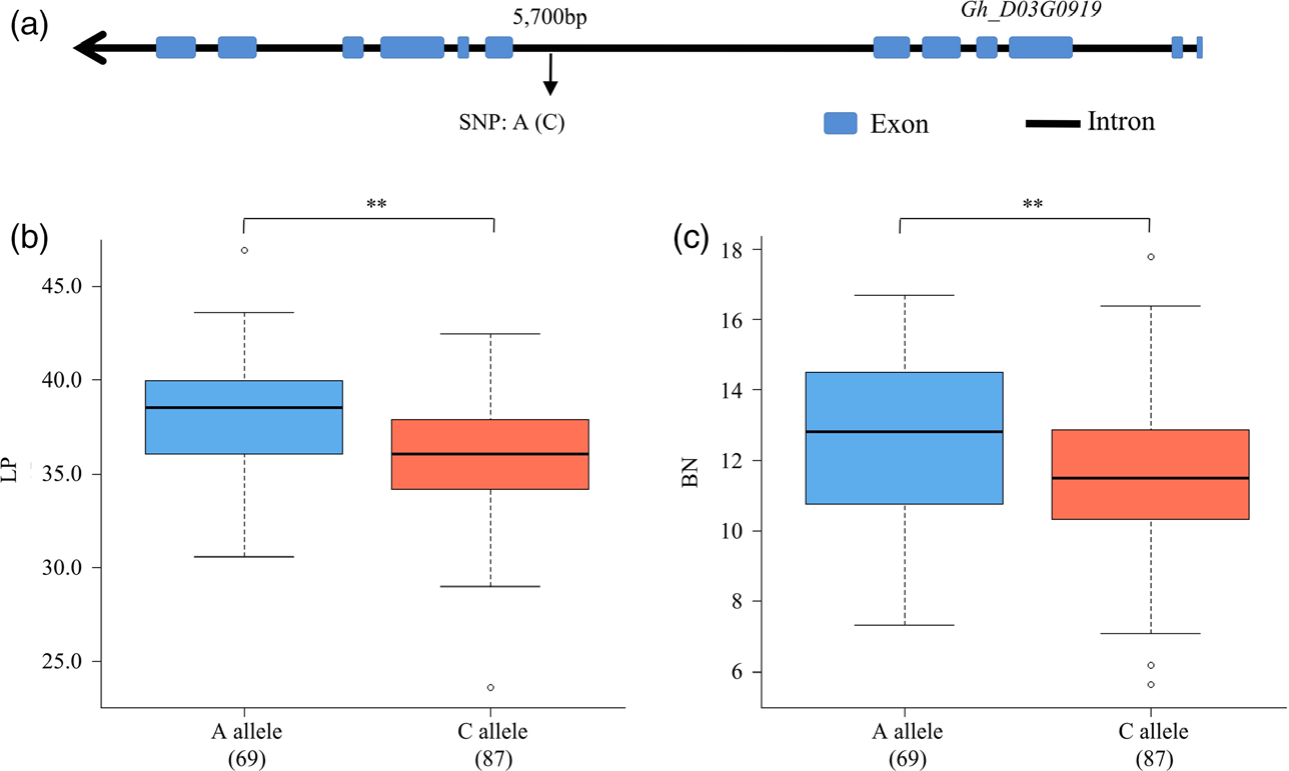
The candidate gene *GhCOBL4* (*Gh_D03G0919*) based association analysis for lint percentage and boll number. (a) Exon–intron structure of *GhCOBL4* and the polymorphism in accessions. (b) Box plots for lint percentage of the two haplotypes. (c) Box plots for boll number of the two haplotypes.

We further investigated these candidate genes using the gene‐based association (GBA) data uploaded by Fang *et al*. (2017b) and found that one SNP (A/C, D3:31243604) at the intron between exon 6 and exon 7 in *Gh_D03G0919* was significantly associated with LP (*P* = 0.000, ANOVA) and BN per plant (*P* = 0.044, ANOVA) (Figure 5a). A total of 156 among 180 genotyped accessions (Table S23) were homozygous at this locus and were divided into two types: AA (69 cultivars) and CC (87 cultivars). The A‐allele type increased the LP and BN per plant by 5.29% and 2.85%, respectively, compared with the C‐allele type (Figure 5b,c).

## Discussion

### SLAF‐based SNP markers are highly effective for constructing high‐density genetic maps

The SLAF‐seq strategy is an effective approach for large‐scale genotyping and construction of a high‐density genetic map (Zhang *et al*., 2015a). In this study, we integrated 3748 SLAF‐based SNP markers and 266 SSR markers to construct a high‐density genetic map. Increasing the marker number not only extends the length of the genetic map but also improves the map resolution (Li *et al*., 2014; Zou *et al*., 2012). In our previous studies, we constructed an SSR‐based genetic map using the same RIL population (Wang *et al*., 2016) that spanned 1943.5 cM with an average interval on each chromosome from 0.65 cM (A05) to 31.1 cM (D04). Using the developed SLAF‐based SNP markers, we improved the map length to 3426.57 cM, and the average distance between adjacent markers on chromosomes was narrowed to an interval of 0.23 cM (A02) to 3.2 cM (D02).

Genetic map construction and GWAS for cotton fibre‐related QTL analysis have also been conducted by using various types of SNP chips (Cai *et al*., 2017; Huang *et al*., 2017; Hulse‐Kemp *et al*., 2015; Liu *et al*., 2018; Sun *et al*., 2017; Tan *et al*., 2018; Zhang *et al*., 2017b); however, chips are expensive because special instruments are needed.

### SLAF‐based high‐density genetic map increases QTL number and narrows QTL interval

It has been reported that the increased marker number and density could enhance the mapped QTL number as well as the precision (Jamshed *et al*., 2016; Li *et al*., 2014; Zou *et al*., 2012). In our previous study, using only SSR markers by the same RIL population, we detected 50 QTLs for fibre‐quality and lint‐yield traits (Wang *et al*., 2016). In the present study, we identified 74 QTLs for all five traits based on the integrated high‐density genetic map, with a 48% increase in QTL number. With a relatively high mapping resolution, 9 of the 24 stable QTLs were mapped to an interval of approximately 1 Mb. This increased number of mapped QTLs and narrowed QTL interval should clearly be attributed to the increased map density achieved with SLAF markers.

### QTL distribution in the A and D sub‐genomes

It has been reported that the D sub‐genome contained significantly more lint‐fibre‐related QTLs than the A sub‐genome (Rong *et al*., 2007), while yield‐related QTLs were almost evenly distributed on the A and D sub‐genomes (Said *et al*., 2013; Yu *et al*., 2013). We found that fibre‐quality QTLs were distributed almost evenly between the two sub‐genomes, while more yield‐related QTLs were located on the D sub‐genome than on the A sub‐genome. This may be because most of the previously reported genetic maps were constructed by using interspecific populations of *G. hirsutum* × *G. barbadense* (Said *et al*., 2015), while one of our mapping parents is an introgression line that contains introgressed chromosomal segments from Sea Island cotton. The continuous selection carried on this parent for simultaneous improvement of fibre quality and yield might result in the observed bias in yield‐related QTL distribution on the two sub‐genomes.

### QTL clusters are possible genetic mechanisms for positive or negative correlations among fibre‐related traits

It has been reported that significant positive correlations exist among the major fibre‐quality traits (Kloth, 1998) and among yield traits (Liu *et al*., 2012), but a significant negative correlation exists between fibre‐quality traits and yield traits, especially LP, a major determinant of lint yield (Wang *et al*., 2011a). The positive correlations are beneficial for the simultaneous improvement of fibre‐quality traits or yield traits, and the negative correlation, known as linkage drag (Young and Tanksley, 1989; Zeven *et al*., 1983), is detrimental because it hinders the simultaneous improvement of these two types of target traits.

QTL clusters indicate that QTLs for various traits co‐localize within the same or adjacent genomic regions on the chromosome, which suggests that pleiotropism or gene linkage may be responsible for positive or negative correlations among traits. QTL clusters associated with fibre‐quality and yield traits have been reported in cotton in various studies (Jamshed *et al*., 2016; Said *et al*., 2015; Su *et al*., 2016; Wang *et al*., 2016). In this study, 12 QTL clusters containing QTLs for fibre‐quality traits as well as for yield traits were identified, and the additive‐effect directions of the QTLs for fibre‐quality and yield traits were opposite except for the QTLs in Cluster‐D10‐1 and Cluster‐D03‐1, indicating that a strong negative correlation existed between fibre quality and yield; this correlation explained why it is difficult to simultaneously improve fibre quality and yield during cotton breeding. However, because the contribution of each QTL in a cluster is different, some of these QTL clusters may still be used in MAS‐based cotton breeding when we consider the trait controlled by the stable QTL as an improvement target. For instance, Cluster‐A07‐1, comprising a lint‐percentage QTL detected in only one environment with a genetic contribution of 3.4% and a QTL for FS with a contribution of 18.55%–37.66% detected in 7 environments, could be used in MAS to improve fibre strength while neglecting the smaller negative effect on yield. Five QTL clusters (Cluster‐A08‐1, Cluster‐D05‐1, Cluster‐D07‐2, Cluster‐D11‐1 and Cluster‐D13‐1) contained only fibre‐quality QTLs, and two (Cluster‐D07‐3 and Cluster‐D07‐5) comprised only yield‐trait QTLs. The directions of the genetic effects of the QTLs in these QTL clusters were similar, suggesting that these QTL clusters could be used to improve fibre‐quality or yield traits.

### Epistasis indicates QTL functions in an interaction network

Epistatic interaction of QTLs has been considered important for understanding the genetic regulation of complex traits (Carlborg and Haley, 2004; Vazquez *et al*., 2015; Yang *et al*., 2010), and recombinant inbred lines (RILs) are recommended for detecting epistatic QTLs (Shang *et al*., 2016). We detected 44 epistatic interactions with additive × additive (AA) effects around the stable QTLs, suggesting that the phenotypic variations of quantitative traits may not be simply explained by the sum of single‐QTL effects (Phillips, 1998). Most of the interactions were detected between stable QTLs and genetic background loci with non‐significant additive effects, which may be due to the limited population size (Peng *et al*., 2011). It was reported that among 156 pairs of epistatic QTLs using the *G*. *hirsutum* RIL population with 196 lines, additive QTLs accounted for <5% (Zhang *et al*., 2017b). Notably, we detected an interaction between two stable QTLs, *qFS‐A07‐1* and *qFS‐D01‐1*, which explained 18.55%–31.96% and 5.15%–10.37% of the phenotypic variation, respectively, implying that *qFS‐A07‐1* may control FS by regulating *qFS‐D01‐1*. *qFS‐A07‐1* was previously reported as a major QTL for FS and had interactions with other QTL loci (Zhang *et al*., 2017b). Some stable QTLs interacted with each other or interacted with more than three loci on different chromosomes, such as *qFS‐A03‐1*, which interacted with 4 loci on Chr. A07, D02, D09 and D12, and *qFU‐A10‐3*, which was an interaction target of three loci on Chr. A03, A05 and A08, suggesting that transcription factors or key genes involved in the same or multiple biological pathways reside in these genomic regions.

### *Gossypium hirsutum* race genetic components contributed more to high fibre quality in LY343

Our sequencing results showed that 94.70% of the genomic sequence of LY343 was from *G. hirsutum* cultivars, 3.88% from *G. hirsutum* races and 1.42% from *G. barbadense*, inconsistent with our earlier conclusions about the derivation of LY343 (Chen *et al*., 2018; Wang *et al*., 2013) and suggesting that LY343 may be derived from a wild cross between *G. hirsutum* and *G. barbadense* followed by several generations of backcrossing to *G. hirsutum* cultivars. The introgressed *G. hirsutum* race segments in LY343 may have been inherited from the *G. barbadense* parent due to the earlier introgression events from *G. hirsutum* races to *G. barbadense* cultivars (Fang *et al*., 2017a; Wang *et al*., 2015).

It was recently reported that a notably long introgressed region from *G. hirsutum* races was present on Chr. A01 of *G. barbadense* and exhibited extremely low levels of SNP polymorphism (Hu *et al*., 2019). We did not find such a long reciprocally introgressed region on any chromosome of LY343. Instead, we detected a long introgressed region from *G. barbadense* that exhibited high levels of SNP polymorphism on Chr. A02 of LY343. However, we found that the majority (76.9%) of favourable alleles were derived from *G. hirsutum* races instead of *G. barbadense* by further analysing the genetic contributions of introgressions to fibre quality. Some important QTL clusters associated with fibre quality, such as Cluster‐A03‐1 and Cluster‐A07‐1, were located in the chromosomal segments introgressed from *G. hirsutum* races, suggesting that the *G. hirsutum* race introgressions may contribute more to the high fibre quality of LY343 than do the *G. barbadense* segments.

### Candidate genes for stable QTLs

The stable QTLs detected in various environments were scarcely affected by the environment, suggesting that key genes exist in those QTL regions. To identify the key genes in the stable QTL regions, we combined DNA resequencing, RNA‐seq and RT‐qPCR data and identified 6 candidate genes for 5 stable QTLs. Two candidate genes encoding pectate lyase 6 (*PEL6*,* Gh_A03G1147*) and cellulose‐synthase‐like C6 (*CSLC6*,* Gh_D07G1598*) were identified for *qFL‐A3‐1* and *qFL‐D7‐1*. The RT‐qPCR results showed that the expression of *GhPEL6* was higher during fibre initiation (0 DPA) and elongation (5 DPA) in LMY22 than in LY343, and *GhCSLC6* expression was higher in the 5‐DPA fibres of LMY22, when the fibres initiate and begin to elongate. It has been reported that the *GhPEL* gene could exclusively degrade de‐esterified pectin and was preferentially expressed during fibre elongation (10 DPA). When the degradation of de‐esterified pectin was repressed by *GhPEL* in the primary cell walls, fibre elongation could be inhibited, which could result in a shortened‐fibre phenotype (Wang *et al*., 2010a). However, no sequence mutation was detected. It is possible either that we cannot detect mutations that exist in this gene or that an unknown regulatory mechanism exists. CSLC6 is a member of the CSLC subfamily, which is responsible for synthesizing the backbone of xyloglucan (Cocuron *et al*., 2007; Liepman and Cavalier, 2012), a primary cell wall hemicellulose. For another fibre‐length QTL, *qFL‐D13‐1*,* GhTBL5* (*Gh_D13G1921*) was identified as a candidate gene. *GhTBL5* was highly expressed in 0 DPA fibres (initiation) in LMY22 and LY343, and there was a significant expression difference between the two parents. It has been reported that some *TBL* gene family members are involved in the cellulose synthesis of secondary walls (Bischoff *et al*., 2010).

Three candidate genes for two lint‐percentage QTLs were identified. *COBRA‐like 4* (*COBL4*,* Gh_D03G0919*), associated with *qLP‐D3‐1*, was highly expressed in 15‐, 20‐ and 25‐DPA fibres in both parents and showed significant differences at 15 and 20 DPA. *COBL4* is essential for cellulose deposition in the secondary cell wall in plants (Li *et al*., 2003). Using the GBA data of a natural population (Fang *et al*., 2017b), we identified one SNP variation in the fifth intron in *GhCOBL4* (*Gh_D03G0919*), and this SNP was significantly related to LP and BN. Although introns do not encode proteins, they have positive effects on gene expression (Gallegos and Rose, 2015; Hir *et al*., 2003).

*MYB4* (*Gh_D09G1659*) and *MYB85* (*Gh_D09G1690*) were identified for *qLP‐D9‐1*, and both were differentially expressed in the developing fibres between LMY22 and LY343. The DNA resequencing data revealed one nonsynonymous SNP mutation in the coding region of *MYB4* (C + 4→G + 4), one indel variation (‐248AATATATATATA‐259) in the promoter region of *MYB4*, and one indel variation (‐68CAAAAAAAAAA‐58) in the promoter region of *MYB85*. The differential expression of *MYB4* and *MYB85* between the two mapping parents may be attributed to indel and/or SNP variation. Both genes are involved in lignin synthesis: *MYB4* negatively regulates lignin synthesis (Jin *et al*., 2000; Wang *et al*., 2011b), but *MYB85* positively regulates lignin synthesis. Overexpression of *MYB85* in Arabidopsis led to lignin deposition in the epidermal and cortical cells of the stems (Zhong *et al*., 2008). However, the candidate genes identified in the current study must be further confirmed by transgenic methods such as overexpression and RNA silencing.

## Experimental procedures

### Plant materials

An intraspecific F_2:8_ RIL population, including 239 lines (Wang *et al*., 2016) and their parents, were planted at Linqing (LQ) in Shandong province from 2011 to 2013, Anyang (AY) in Henan province and Lishui (LS) in Jiangsu province in 2012 and 2013. The planting dates and densities were described in our previous study (Wang *et al*., 2016). All trials were laid out in a completely randomized design with two replications. Each test location and year combination was considered an individual environment, and abbreviations were used to distinguish the different environments; that is LQ11, LQ12, LQ13, AY12, AY13, LS12 and LS13 represented Linqing in 2011, Linqing in 2012, Linqing in 2013, Anyang in 2012, Anyang in 2013, Lishui in 2012 and Lishui in 2013, respectively.

### Phenotypic evaluation

Twenty fully opened inner bolls from the middle fruit branches were harvested in all seven environments for investigating fibre quality and yield traits. Six representative plants were tagged and investigated in three environments (LQ11, AY12 and AY13) for BN per plant. The fibre‐quality parameters were tested by the Supervision, Inspection and Test Center of Cotton Quality, Ministry of Agriculture of China (Anyang, Henan province) using a high‐volume precision instrument. BN, BW and LP were evaluated by conventional cotton breeding methods. Phenotypic variations and correlation analysis were calculated using SPSS version 17.0 software.

### SLAF library construction

Genomic DNA of 239 RILs together with their parents was extracted as described by Paterson *et al*. (1993). SLAF library construction was performed as described by Sun *et al*. (2013) with some modifications. First, a pre‐experiment enzyme digestion was conducted based on the reference genome of *G. hirsutum* acc. TM‐1 (Zhang *et al*., 2015b), and two endonucleases, HaeIII and SspI (New England Biolabs, NEB, USA), were used to digest the genomic DNA of our RIL population. DNA fragments of approximately 500 bp were selected as SLAFs and prepared for paired‐end sequencing on an Illumina HiSeq 2500 sequencing platform at Biomarker Technologies Corporation (Beijing, China).

### SLAF‐seq grouping and genotyping

The SLAF‐seq data were grouped and genotyped as described by Sun *et al*. (2013). First, the low‐quality reads (quality score < 20e) were filtered out, and the raw reads were trimmed according to the predefined criteria. Finally, 80‐bp paired length sequences were retained at each end, and then, these clean reads were mapped onto the reference genome of *G. hirsutum*acc. TM‐1 using BWA software (Li and Durbin, 2009). The SLAF groups were generated by reads mapped to the same position. Sequences mapped to the same position with over 95% identity were grouped into one SLAF locus. By comparing the sequence of the RILs together with their parents, all SLAF tags were divided into three types: polymorphic, repetitive and non‐polymorphic. The repetitive and non‐polymorphic SLAFs were discarded, and the polymorphic SLAFs were retained for genotyping according to the consistency of SNP loci in the parents and offspring.

### Genetic map construction

The efficient SLAF markers and 226 polymorphic SSRs (Wang *et al*., 2016) were first mapped to 26 linkage groups (LGs) based on the *G. hirsutum* reference genome. The HighMap software (Liu *et al*., 2014) was used to construct a genetic map within each of the LGs with MLOD scores > 5. The Kosambi mapping function was used to calculate genetic map distance (centiMorgans, cM). The collinearity of the LGs with the TM‐1 reference genome was compared using the local Basic Local Alignment Search Tool (BLAST). Any region containing more than three consecutive skewed (*P* < 0.05) loci was defined as an SDR.

### QTL mapping and epistasis analysis

The phenotype data of five fibre‐quality parameters and three yield elements collected from all seven environments were prepared for QTL mapping. WinQTLCart 2.5 was applied to detect QTLs in each individual environment based on composite interval mapping (CIM) (Wang *et al*., 2012). The window size was set at 5 cM, and the walk speed was 1 cM. LOD threshold values were estimated by 1000 permutations to declare significant QTLs. QTLs detected in more than three environments were defined as stable QTLs. However, QTLs for BN per plant, detected in two or more environments, were identified as stable QTLs due to its phenotype data collected from only three environments. QTL nomenclature referred to the method developed by Mccouch *et al*. (1997). The designation begins with ‘q’, followed by an abbreviation of the trait, chromosome and the serial number. QTL interactions were identified using the IciMapping v3.3 software (Meng *et al*., 2015).

### Genomic component analysis

A ‘three‐population test’ method (Fang *et al*., 2017a; Myles *et al*., 2011) was used to analyse the genetic components of LY343. Briefly, we used the deep‐sequencing data of LY343 (NCBI Sequence Read Archive (SRA) with the accession number PRJNA546484) together with the genomic data of 147 cotton accessions (Fang *et al*., 2017a) to construct a phylogenetic tree based on the SNPs in every 500‐Kb genomic sequence on the 26 chromosomes. We investigated each of the phylogenetic trees to identify the genetic components of LY343 based on which clade LY343 was divided into, and finally, we determined whether each genomic components was derived from *G. hirsutum* cultivars, *G*. *hirsutum* races or *G. barbadense*.

### Candidate QTL interval determination

We examined the intervals of stable QTLs, as they were less affected by the environment, and 99% confidence intervals of a stable QTL in various environments were determined as candidate regions. Genes located in the candidate regions were examined by referring to the TM‐1 genome data (Zhang *et al*., 2015b). The genes related to fibre development were selected for further analysis.

### Gene functional annotation

Gene Ontology and KEGG analyses of candidate genes in the QTL clusters were performed on the Cotton Functional Genomics Database (CottonFGD) (https://cottonfgd.org/) (Zhu *et al*., 2017). Expression profiles of candidate genes were analysed using the R package mfuzz and visualized using the R software pheatmap.

### RNA isolation, RNA‐Seq and RT‐qPCR

More than three biological replications of ovules with fibres at 0 and 5 DPA and fibres at 10, 15, 20 and 25 DPA of LMY22 and LY343 were collected from the field during fibre development. Total RNA was extracted using a Plant RNA Purification Kit (Tiangen, Beijing, China) following the manufacturer's instructions. The RNA samples of two biological replications at various fibre developmental stages from both parents were sent to Novogene Bioinformatics Technology Co., Ltd. (Beijing, China) for RNA sequencing using the HiSeqXten platform. The gene expression level was calculated using fragments per kilobase of exon model per million mapped reads (FPKM) with Cufflinks (version 2.1.1) (Trapnell *et al*., 2010).

Total RNA from three biological replicates at each stage of fibre development, as described above, was converted to cDNA using a PrimeScript RT reagent kit with gDNA Eraser (TaKaRa) and subjected to reverse transcription quantitative polymerase chain reaction (RT‐qPCR). The primer sequences for RT‐qPCR were designed using Primer 5.0 software. Relative expression levels of candidate genes were calculated using the 2^−∆∆Ct^ method (Livak and Schmittgen, 2001). *GhActin9* was used as an internal control to normalize sample variance.

### SNP and indel annotation

The DNA resequencing data of two mapping parents were from our laboratory (NCBI accession number PRJNA546484). Briefly, the genomic DNA of both parents was extracted using the CTAB protocol. The qualified DNA samples were sent to Annoroad Gene Technology (Beijing, China) for sequencing on the Illumina HiSeqXten platform. A total of 684.4 M reads were generated, with each read 150 bp in size and an average 40 × genome coverage for each parent. SNP and indel calling were performed using the Genome Analysis Toolkit (GATK) (Mckenna *et al*., 2010) HaplotypeCaller protocol via local re‐assembly of haplotypes for accessions. SNPs and indels were annotated using ANNOVAR (Wang *et al*., 2010b) for all qualified variants based on the GFF file.

A natural population containing 180 upland cotton accessions was randomly chosen for evaluating the effect of allelic variation of *GhCOBL4* (*Gh_D03G0919*), the candidate gene of *qLP‐D3‐1*.

## Conflict of interest

The authors declare that the research was conducted in the absence of any commercial or financial relationships that could be construed as a potential conflict of interest.

## Author contributions

JZ, FRW, SJF and YLY designed the experiments. FRW, JXZ, YC, CYZ, JWG, JZ, JJW, AYL and ZHD performed field trials, phenotypic evaluation and data collection. YC and JXZ, JJW, CJZ, MJJ, FRW and JZ contributed to the preparation of cotton DNA samples and performed the analysis of QTL mapping data, with the help of FRW for the analysis of raw data. YC and FRW contributed to *in silico* bioinformatic analysis. ZQS performed the preparation of cotton RNA samples and RT‐qPCR. FRW drafted the manuscript. JZ, SJF and YLY revised the manuscript. All authors read and approved the final manuscript.

## Supporting information


**Figure S1** Construction of genetic map by SLAF markers. (a) Distribution of polymorphic SLAF markers on each of the 26 chromosomes. The black vertical lines on the chromosomes indicate SLAF markers. (b) Collinearity analysis of 26 linkage groups with the TM‐1 reference genome. The *x*‐axis represents linkage group number of genetic map and the *y*‐axis indicates the chromosome number of TM‐1 reference genome.

**Figure S2** A phylogenetic tree of LY343 and 147 cotton accessions including *Gossypium hirsutum* races, *G. hirsutum* cultivars and *G. barbadense* cultivars.

**Figure S3** Functional annotation of the expressed genes in 17 QTL clusters. (a) Go analysis of candidate genes. Only 36 terms with more than 10 genes in biological process were shown. (b) KEGG analysis of candidate genes. The pathways associated only with fibre development were showed. (c) Heat map of genes enriched in cell wall organization or biogenesis, macromolecule localization and microtubule‐based process.

**Figure S4** Annotation of genes in expression profile of Cluster 3.

**Figure S5** Annotation of genes in expression profile of Cluster 2.

**Figure S6** Annotation of genes in expression profile of Cluster 6.

**Figure S7** Annotation of genes in expression profile of Cluster 1.

**Figure S8** Annotation of genes in expression profile of Cluster 10.

**Figure S9** Relative expression level of genes downstream of *MYB4* (a) and *MYB85* (b).

**Table S1** Summary of SLAF‐seq data.

**Table S2** Distribution of SLAF markers on 26 chromosome of upland cotton.

**Table S3** Basic characteristics of high‐density genetic map.

**Table S4** Marker position in genetic map and physical map.

**Table S5** Collinearity analysis of the genetic map with the physical map.

**Table S6** Phenotypic variations of fibre‐quality and yield traits in RIL population.

**Table S7** Correlation coefficients among fibre‐quality and yield traits.

**Table S8** QTLs detected based on high‐density genetic map and comparison with our previously reported QTLs.

**Table S9** Stable QTLs detected in multiple environments.

**Table S10** Epitasis of QTLs for fibre‐quality and yield traits.

**Table S11** QTL clusters identified for fibre‐quality and yield traits.

**Table S12** Summary of introgression components from *G. barbadense* and *G. hirsutum* race in LY343.

**Table S13** Introgression components from *G. barbadense*.

**Table S14** Introgression components from *Gossypium hirsutum* race.

**Table S15** QTL overlapping with introgression chromosome segment.

**Table S16** QTL clusters overlapping with the introgressed segments.

**Table S17** 2441 genes in 17 QTL clusters.

**Table S18** Go annotation information of candidate genes in 17 QTL clusters.

**Table S19** KEGG annotation information of the candidate genes in the QTL clusters.

**Table S20** Expression profile of candidate genes in 17 QTL clusters.

**Table S21** The corresponding interval of some stable QTLs in TM‐1 reference genome.

**Table S22** SNP and indel variation in upstream and coding region of candidate genes.

**Table S23** Cotton accessions for allelic variation evaluation of *Gh_D03G0919*.
