# Supplementary figures and images for: Identification of candidate genes for key fibre‐related QTLs and derivation of favourable alleles in Gossypium hirsutum recombinant inbred lines with G. barbadense introgressions
Source: Plant Biotechnol J. 2019 Sep 20;18(3):707–20. doi: 10.1111/pbi.13237 (PMC7004909; doi:10.1111/pbi.13237)

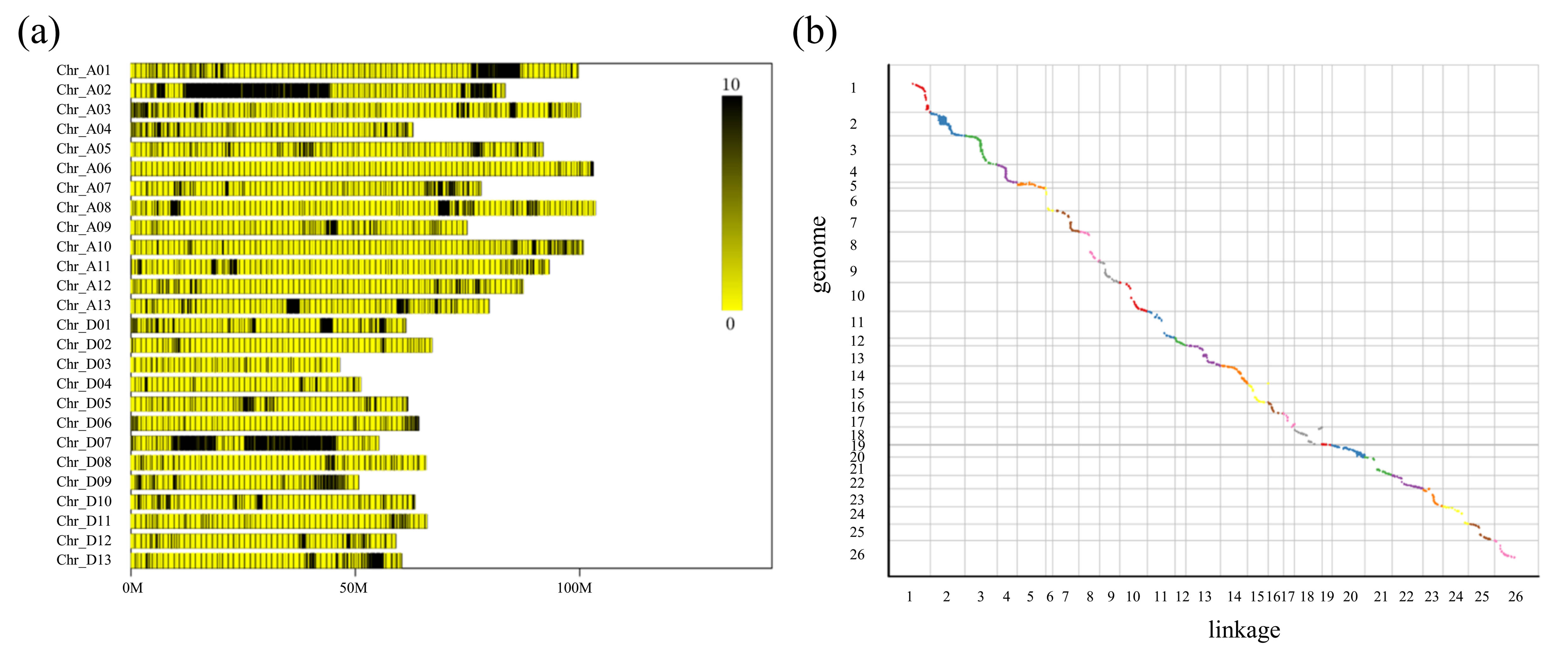

Supplement: Supplementary file 1 — Figure S1 Construction of genetic map by SLAF markers. (a) Distribution of polymorphic SLAF markers on each of the 26 chromosomes. The black vertical lines on the chromosomes indicate SLAF markers. (b) Collinearity analysis of 26 linkage groups with the TM‐1 reference genome. The x‐axis represents linkage group number of genetic map and the y‐axis indicates the chromosome number of TM‐1 reference genome. [file PBI-18-707-s001.jpg]

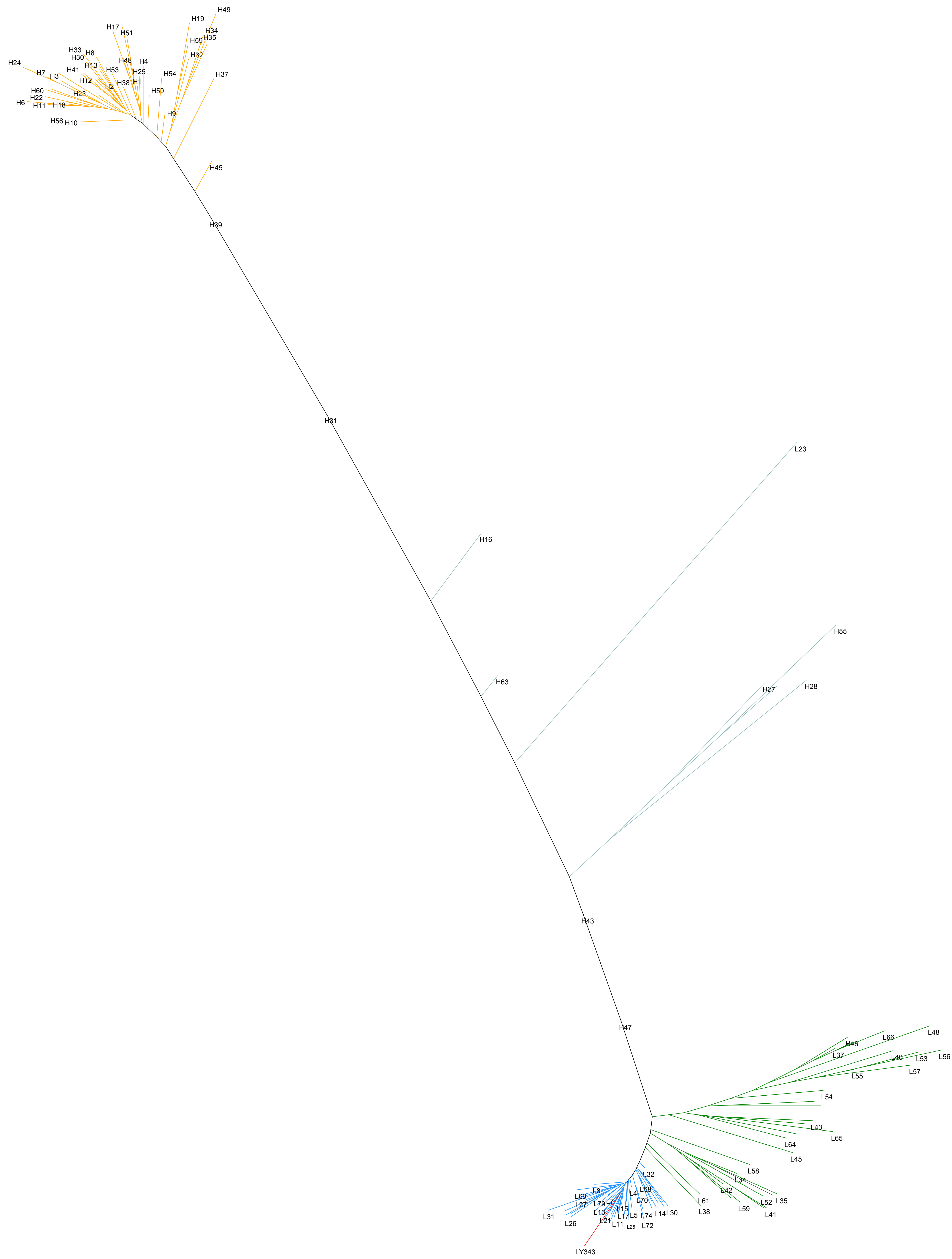

Supplement: Supplementary file 2 — Figure S2 A phylogenetic tree of LY343 and 147 cotton accessions including Gossypium hirsutum races, G. hirsutum cultivars and G. barbadense cultivars. [file PBI-18-707-s030.pdf]

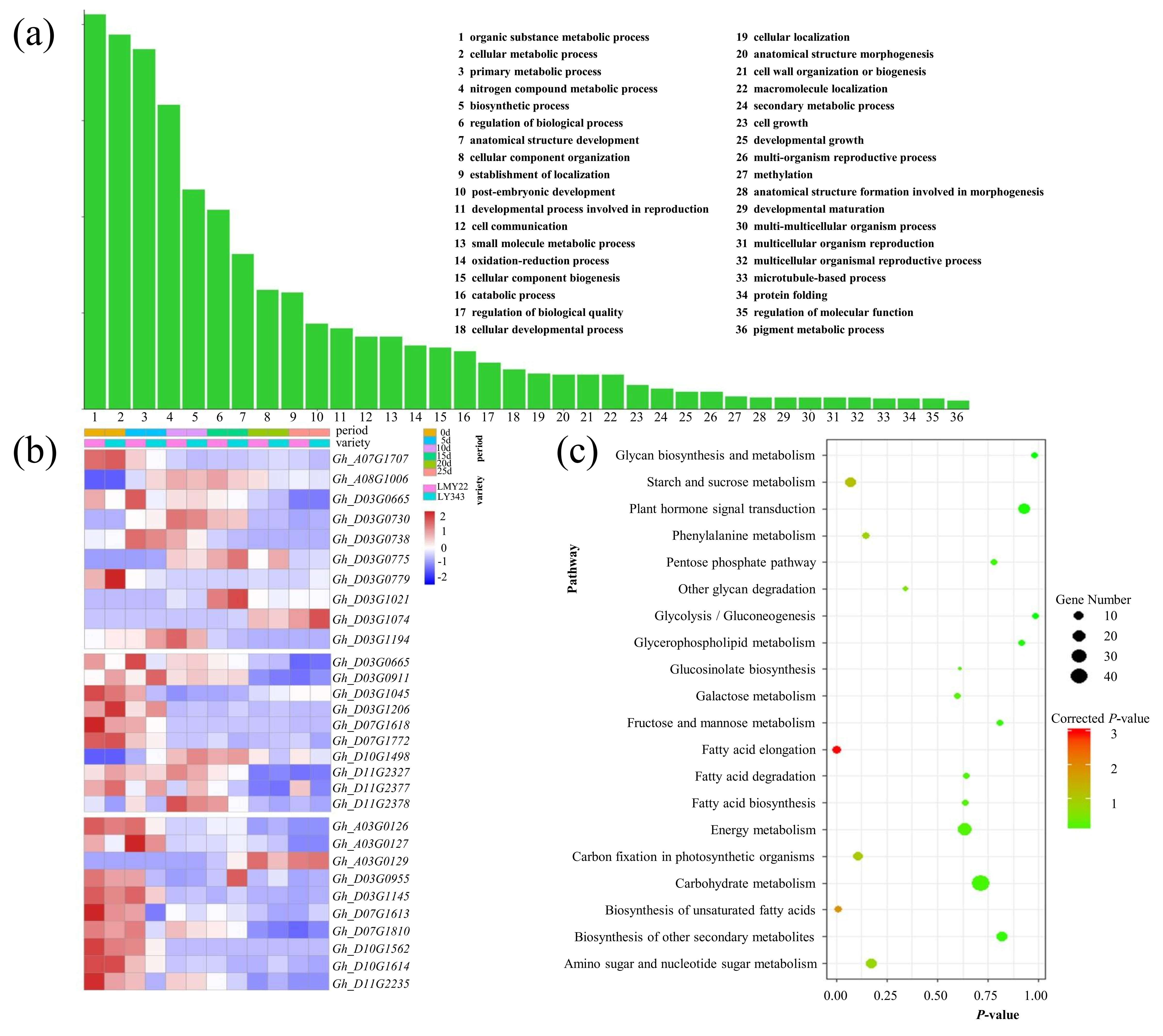

Supplement: Supplementary file 3 — Figure S3 Functional annotation of the expressed genes in 17 QTL clusters. (a) Go analysis of candidate genes. Only 36 terms with more than 10 genes in biological process were shown. (b) KEGG analysis of candidate genes. The pathways associated only with fibre development were showed. (c) Heat map of genes enriched in cell wall organization or biogenesis, macromolecule localization and microtubule‐based process. [file PBI-18-707-s029.jpg]

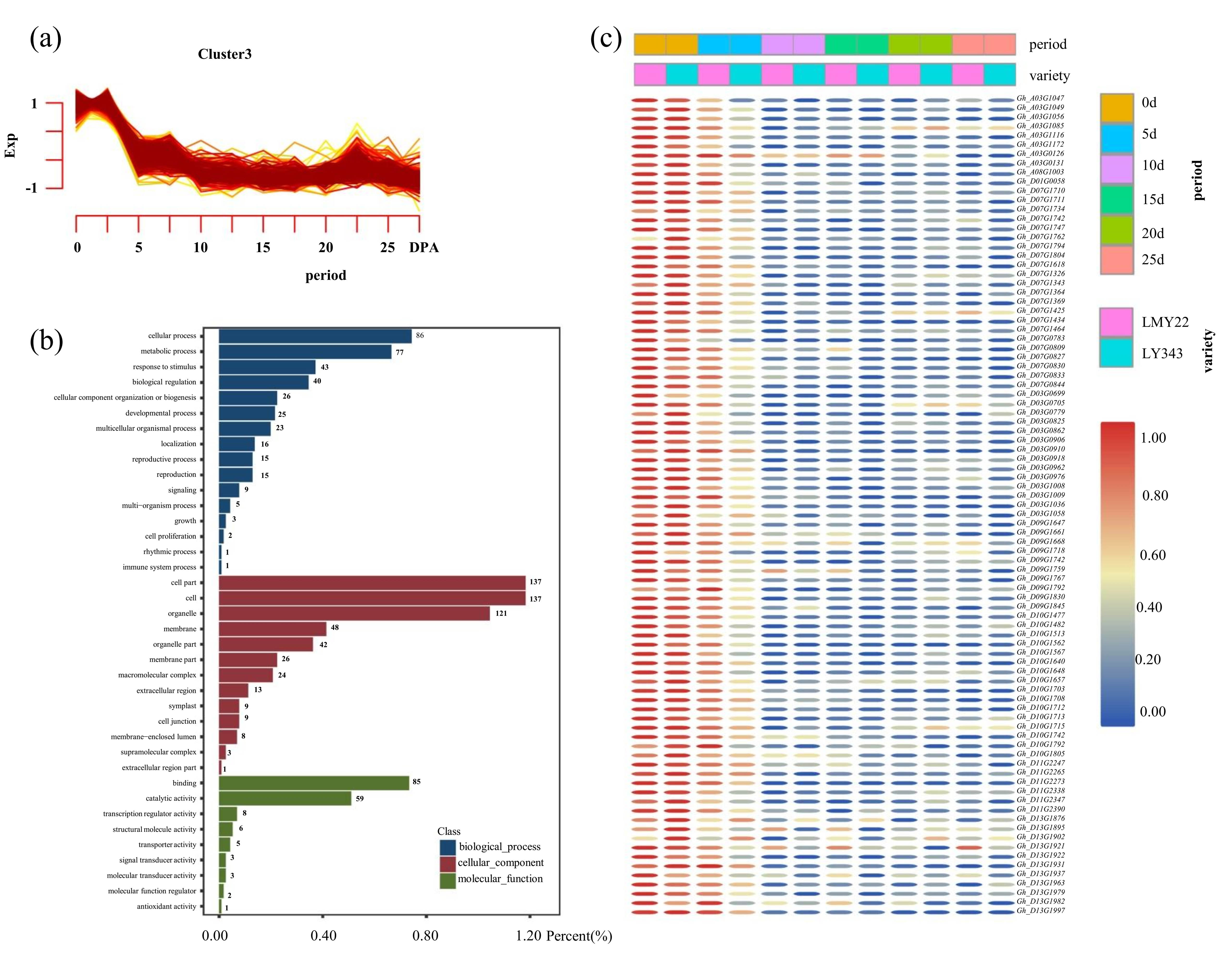

Supplement: Supplementary file 4 — Figure S4 Annotation of genes in expression profile of Cluster 3. [file PBI-18-707-s031.jpg]

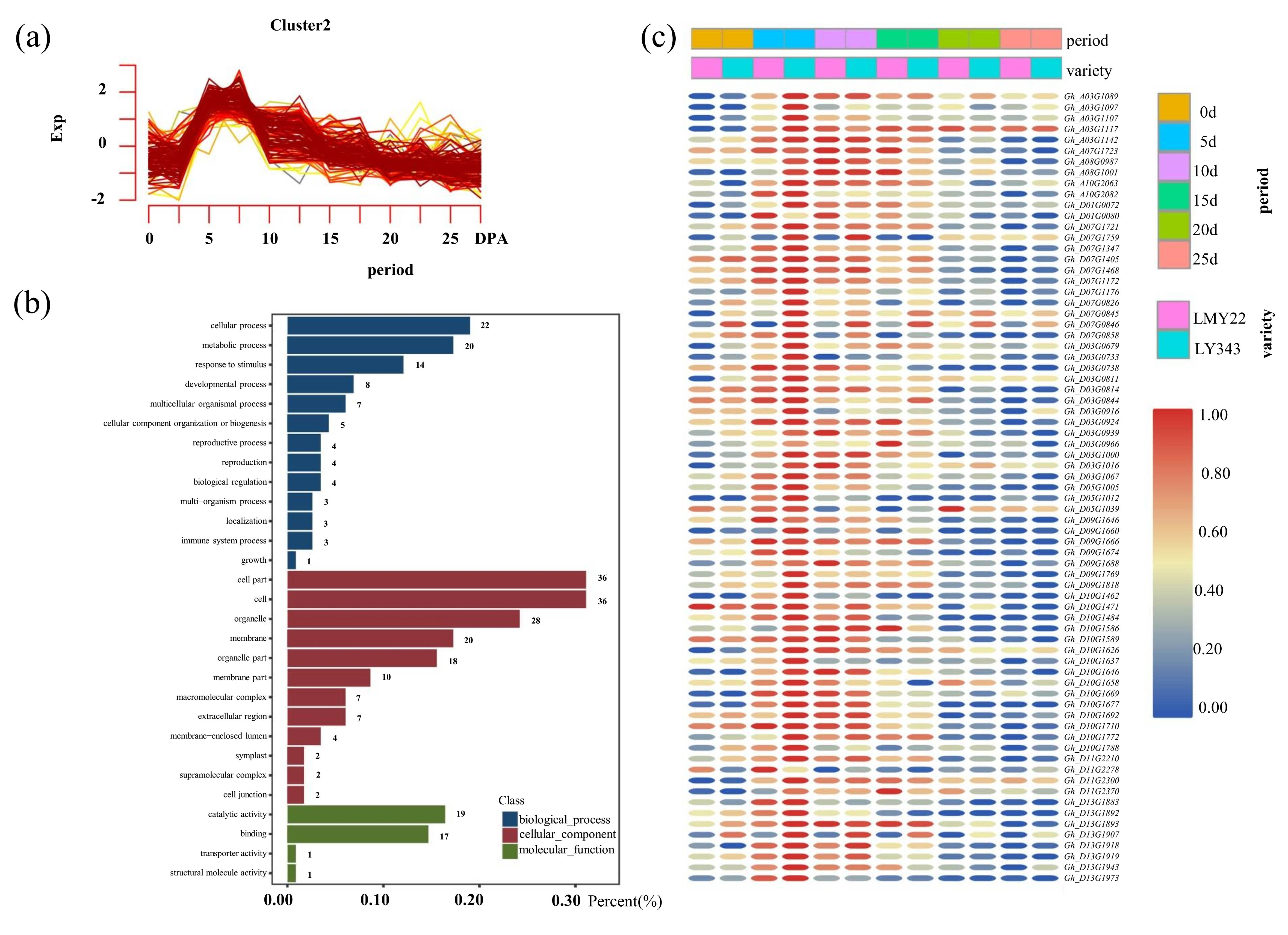

Supplement: Supplementary file 5 — Figure S5 Annotation of genes in expression profile of Cluster 2. [file PBI-18-707-s032.jpg]

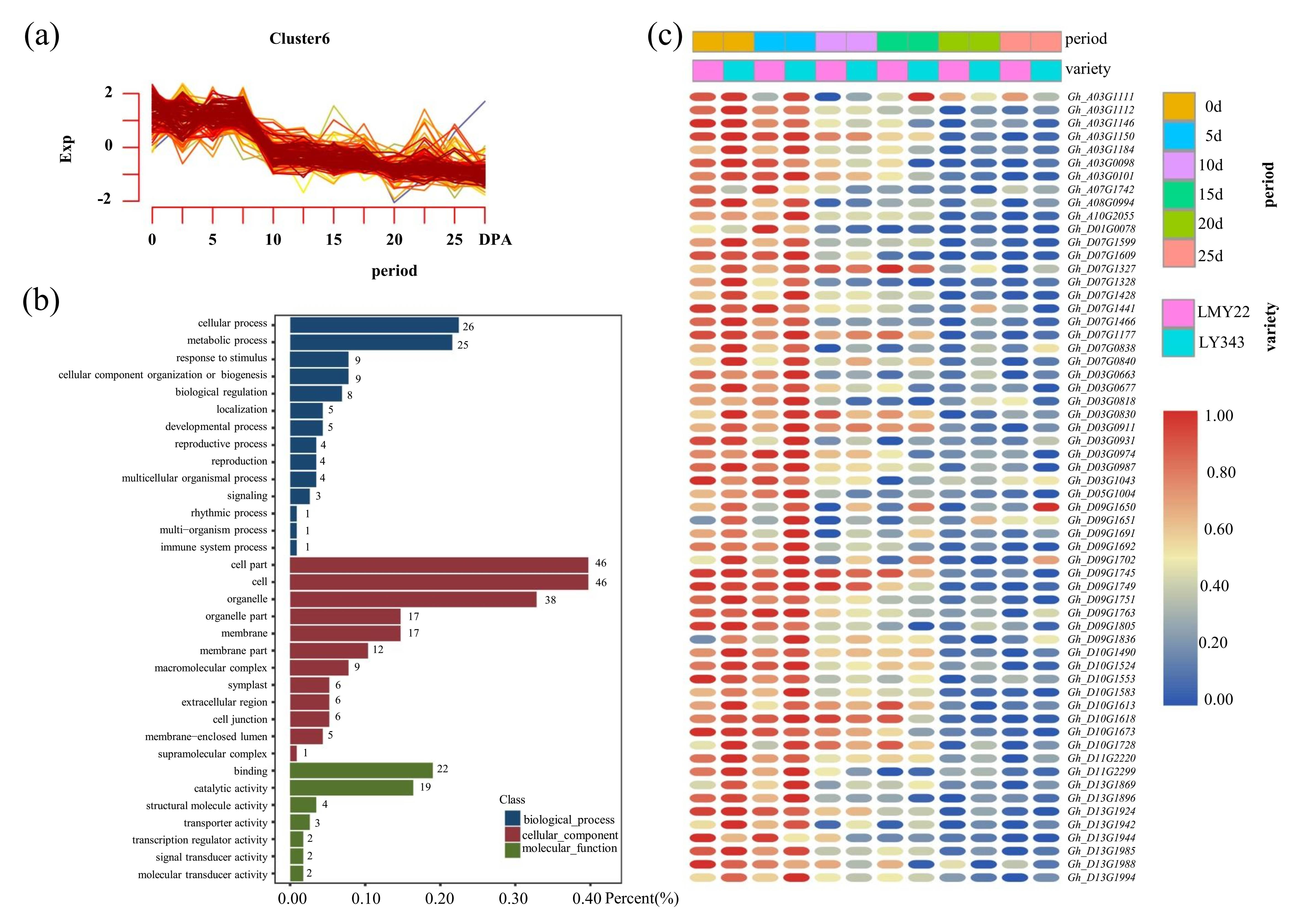

Supplement: Supplementary file 6 — Figure S6 Annotation of genes in expression profile of Cluster 6. [file PBI-18-707-s002.jpg]

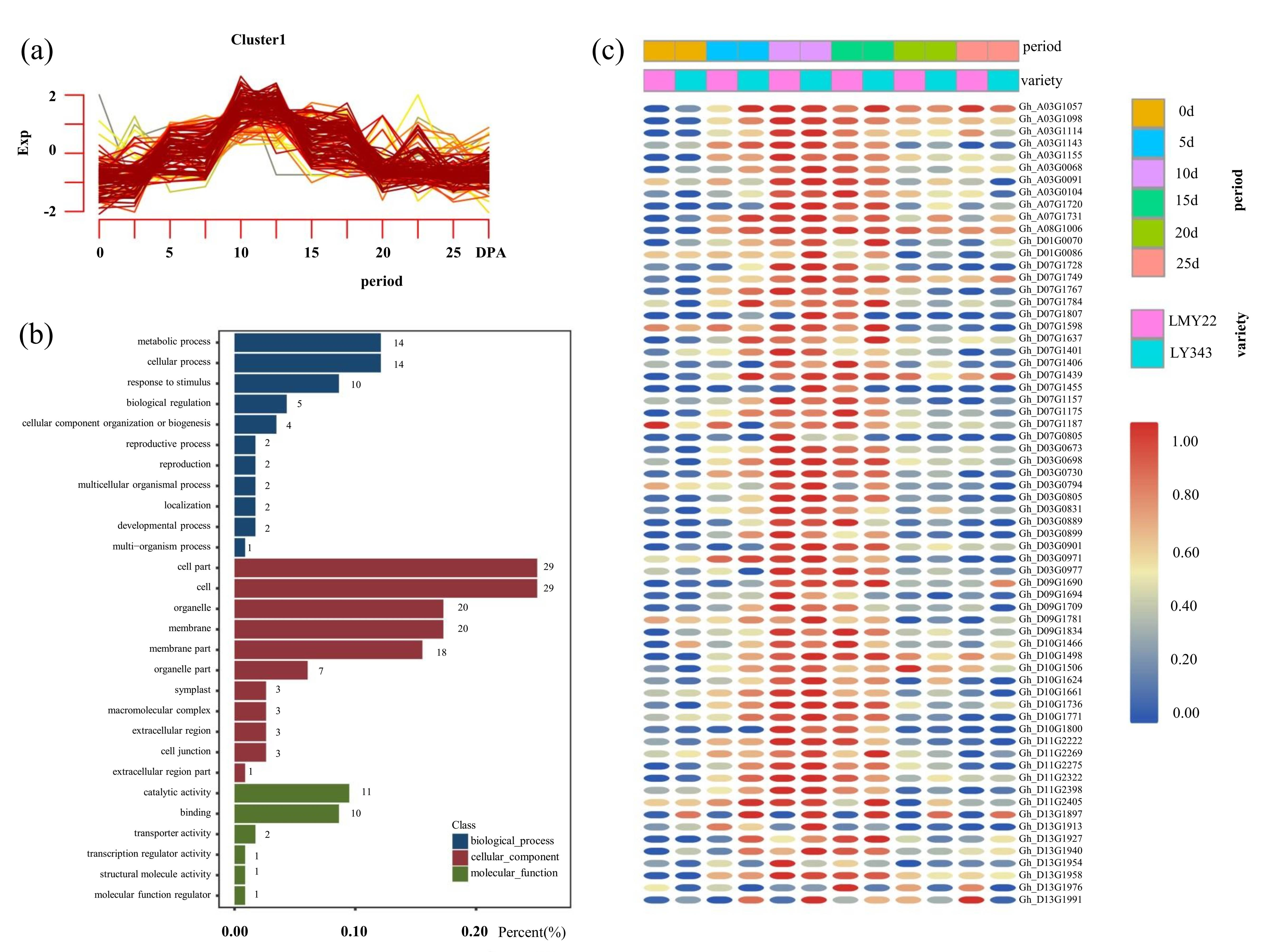

Supplement: Supplementary file 7 — Figure S7 Annotation of genes in expression profile of Cluster 1. [file PBI-18-707-s003.jpg]

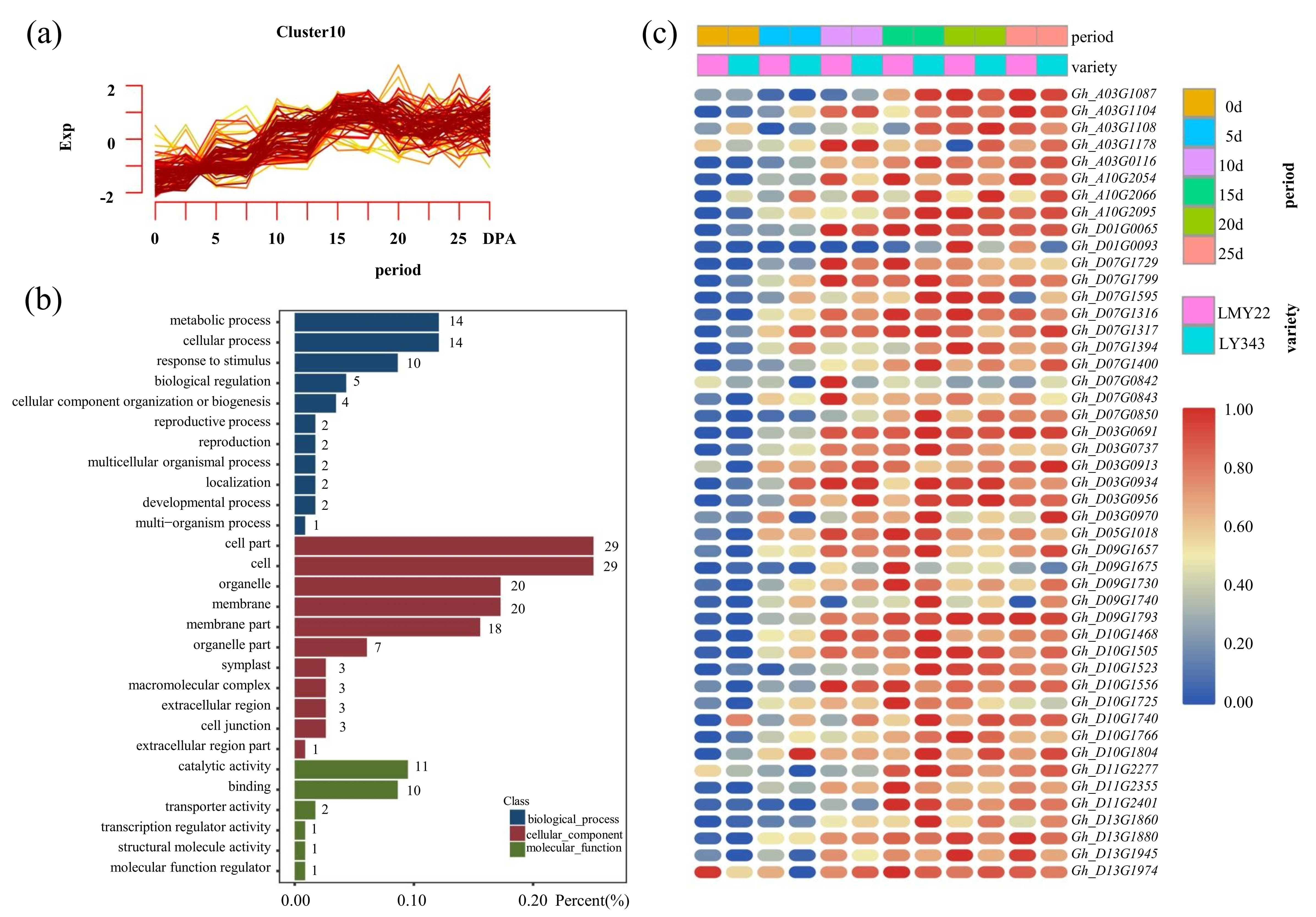

Supplement: Supplementary file 8 — Figure S8 Annotation of genes in expression profile of Cluster 10. [file PBI-18-707-s004.jpg]

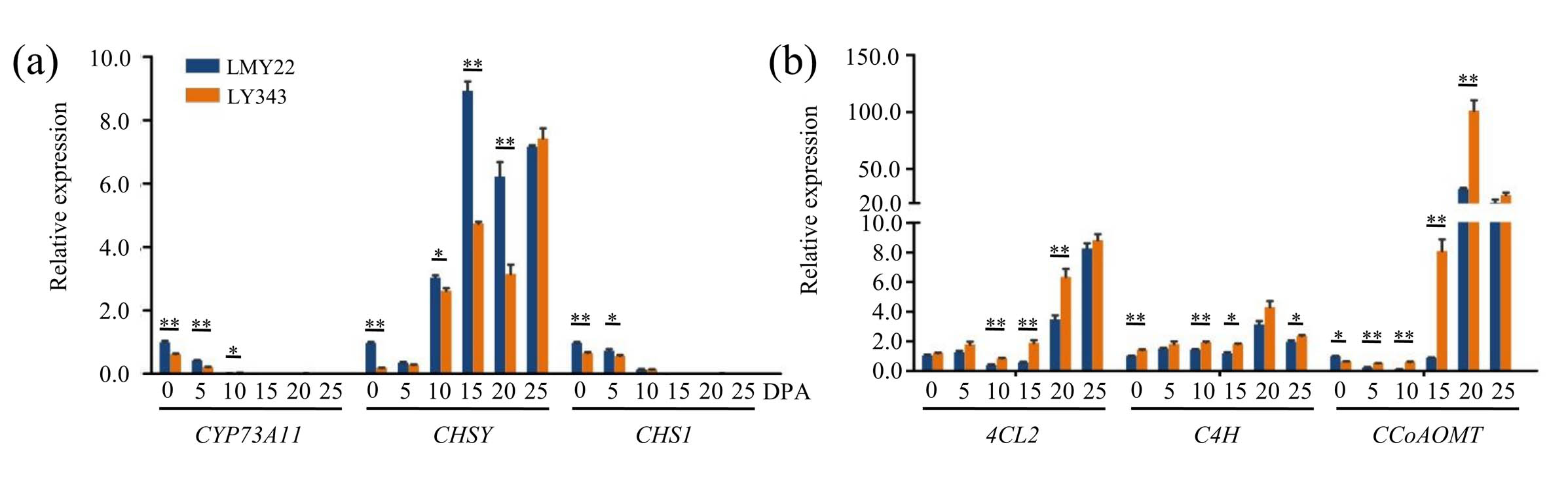

Supplement: Supplementary file 9 — Figure S9 Relative expression level of genes downstream of MYB4 (a) and MYB85 (b). [file PBI-18-707-s005.jpg]
